# Efficacy and Safety of First-Line Everolimus Therapy Alone or in Combination with Octreotide in Gastroenteropancreatic Neuroendocrine Tumors. A Hellenic Cooperative Oncology Group (HeCOG) Study

**DOI:** 10.3390/biology9030051

**Published:** 2020-03-09

**Authors:** Anna Koumarianou, Dimitrios Pectasides, Georgia-Angeliki Koliou, Dimitrios Dionysopoulos, Dionysia Kolomodi, Christos Poulios, Maria Skondra, Joseph Sgouros, George Pentheroudakis, Gregory Kaltsas, George Fountzilas

**Affiliations:** 1 Hematology Oncology Unit Fourth Department of Internal Medicine, National and Kapodistrian University of Athens, Medical School, Attikon University Hospital, 12462 Athens, Greece; 2 Oncology Section, Second Department of Internal Medicine, Hippokration Hospital, 11527 Athens, Greece; pectasid@otenet.gr (D.P.); rikaskondra@gmail.com (M.S.); 3 Section of Biostatistics, Hellenic Cooperative Oncology Group, Data Office, 11526 Athens, Greece; g_koliou@hecog.ondsl.gr; 4 Department of Medical Oncology, Papageorgiou Hospital, Aristotle University of Thessaloniki, School of Health Sciences, Faculty of Medicine, 56403 Thessaloniki, Greece; dimitris.dionysopoulos@gmail.com; 5 First Department of Propaedeutic Internal Medicine, National and Kapodistrian University of Athens, Medical School, Laiko University Hospital, 11527 Athens, Greece; denisekolomodi@hotmail.gr (D.K.); gregory.kaltsas@gmail.com (G.K.); 6 Department of Pathology, Aristotle University of Thessaloniki, School of Health Sciences, Faculty of Medicine, 54006 Thessaloniki, Greece; dr.poulios@hotmail.com; 7 Third Department of Medical Oncology, Agii Anargiri Cancer Hospital, 14564 Athens, Greece; josephsgouros@yahoo.co.uk; 8 Department of Medical Oncology, Medical School, University of Ioannina, 45110 Ioannina, Greece; gpenther@otenet.gr; 9 Society for Study of Clonal Heterogeneity of Neoplasia (EMEKEN), 45110 Ioannina, Greece; 10 Aristotle University of Thessaloniki, 54006 Thessaloniki, Greece; fountzil@auth.gr; 11 Laboratory of Molecular Oncology, Hellenic Foundation for Cancer Research/Aristotle University of Thessaloniki, 54006 Thessaloniki, Greece; 12 Department of Medical Oncology, German Oncology Center, Limassol 4108, Cyprus

**Keywords:** GEP NET, targeted therapy, octreotide LAR, liver, lymph node, metastases

## Abstract

The purpose of this study was to explore the efficacy and safety of everolimus administered as a first-line treatment in newly diagnosed patients with metastatic or inoperable gastroenteropancreatic neuroendocrine tumors (GEP NETs). This phase II, multicenter, single-arm study included patients with well-differentiated GEP NETs and a Ki67 < 20%. Everolimus, at 10 mg/day, was administered until disease progression; 18 patients (72%) concomitantly received octreotide long-acting release (LAR), at 30 mg/month. The primary endpoint was the 15-month progression-free survival (PFS) rate. Twenty-five patients (grade 1: 11 patients, grade 2: 14 patients) were enrolled between August 2012 and October 2015. At a median follow-up of 58.1 months, the median PFS was 14.6 months, while the 15-month PFS rate was 48%; median overall survival had not been reached yet. Normal baseline chromogranin A (<4 nmol/l) confirmed a longer PFS (HR = 0.25, 95% CI 0.08–0.77, *p* = 0.016). Seven patients (28%) achieved an objective response (one complete response and six partial responses) in a median of 2.6 months. Twenty-three grade 3–4 events were recorded (14 patients). No fatal reactions occurred. This prospective phase II study unravels the notable activity of everolimus as a first-line treatment in patients with GEP NETS and contributes valuable information about the high activity of the combination of everolimus and octreotide LAR in this setting. Clinical trial information: NCT01648465.

## 1. Introduction

Gastroenteropancreatic neuroendocrine tumors (GEP NETs), or as more recently called, gastrointestinal neuroendocrine neoplasms (GI NENs), are derived from the diffuse endocrine system and can cause symptoms due to either mass effects on surrounding tissues (non-functioning NENs) or symptoms secondary to the secretion of bioactive compounds (peptides and amines) that lead to distinct clinical syndromes (functioning NENs) [1]. Most of these neoplasms are well-differentiated, expressing on their cell surface somatostatin receptors: a feature used for both their diagnosis and treatment.

According to the Surveillance, Epidemiology, and End Results (SEER) Program—a large epidemiological study conducted in the USA—the incidence of NETs has increased from 3.96 in 1995 to 6.61 in 2011, most probably due to improved diagnostic modalities and histopathological classification systems [2]. The most recent SEER database analyses indicated significant improvements in the overall survival (OS) of patients with metastatic GEP NETs in the last decade [3]. This benefit was attributed by the authors to increased awareness and the recently approved targeted systemic therapies, such as long-acting somatostatin analogs (octreotide long-acting release (LAR) and lanreotide Autogel), sunitinib, and everolimus [4,5,6,7]. However, further improvement in the therapeutic field is required, as the same study has indicated that the median OS of patients with metastatic NETs, irrespective of grade, remains relatively low at 12 months and ranges from 5.83 years for NETs that occur in the small intestine, to 6 and 4 months for NETs that occur in the lung and colon, respectively [3].

Somatostatin analogs bind to G-protein-linked receptors on the cell surface and cause increased apoptosis, cell cycle arrest, and the inhibition of cell invasion and angiogenesis [8]. The clinical efficacy of somatostatin analogs has been shown in two phase III trials and one meta-analysis that confirmed a reduction in disease progression in advanced GEP NETs by 41%, with a good safety profile [4,9,10]. Gastroenteropancreatic tumors are characterized by the activation of the mammalian target of rapamycin (mTOR) pathway, which is a major regulator of protein synthesis, integrating the mitogenic signals elicited by growth factors and the availability of resources, and controlling the initiation stage of mRNA translation and ribosome biogenesis [11]. Everolimus, an oral inhibitor of mTOR, has shown clinical activity as a single agent in patients with advanced progressive well- and moderately-differentiated GEP NETs, in three randomized phase III studies: the RADIANT−2, −3, and −4 [6,7,12].

Based on their mechanism of action, the combination of somatostatin analogs with mTOR inhibitors may provide synergistic effects for the treatment of patients with GEP NETs [13]. Three clinical studies have suggested that the combined use of octreotide and everolimus could achieve an increase in clinical benefits compared to the use of either of the agents [12,14,15].

The aim of this study was to test the clinical activity of everolimus as a frontline therapy in the treatment of naïve patients with advanced GEP NETs. The co-administration of octreotide LAR at study entry was allowed upon the physician’s decision.

## 2. Patients and Methods

The protocol was approved by the National Organization for Medicines (EOF), the Institutional Review Board, or Ethics Committee at each participating center, and the study was conducted in accordance with Good Clinical Practice Principles, the applicable local regulations and the World Medical Association Declaration of Helsinki. All patients provided written informed consent prior to treatment initiation. The study was designed by AK and by representatives of the sponsor.

### 2.1. Treatment Protocol

This phase II study included patients with treatment-naïve, metastatic, low grade GEP NETs (Ki67 < 20%). Patients were treated with everolimus at 10 mg/day as a first-line therapy until progressive disease (PD) or unacceptable toxicity. Based on the physician’s choice, patients could be treated with somatostatin analogs in addition to everolimus as the first-line treatment. The primary endpoint of the study was the 15-month Progression Free Survival (PFS) rate, as evaluated by RECIST 1.1 criteria. The secondary endpoints included PFS; OS; the safety profile (including serious and non-serious adverse events); the best response rate (RR), including the objective response rate (ORR) = Complete Response (CR), the Partial Response (PR), and the disease control rate (DCR) = CR+PR+Stable Disease (SD); and the time to best response (TTR).

The inclusion criteria included 1. a newly diagnosed metastatic biopsy-proven neuroendocrine tumor; 2. measurable disease using RECIST criteria on a Computed Tomography (CT) scan or Magnetic Resonance Imaging (MRI); 3. adequate bone marrow function as shown by ANC ≥ 1.5 x 10^9^/L, platelets ≥ 100 x 10^9^/L, and Hb > 9 g/dL; 4. adequate liver function as shown by serum bilirubin ≤ 1.5 x ULN, INR < 1.3 x ULN (or < 3 on anticoagulants), and ALT and AST ≤ 2.5x ULN (≤ 5x ULN in patients with liver metastases); 5. adequate renal function: serum creatinine ≤ 1.5 x ULN; 6. fasting serum cholesterol ≤ 300 mg/dL or 7.75 mmol/L, and fasting triglycerides ≤ 2.5 x ULN (in case one or both of these thresholds were exceeded, the patient could only be included after the initiation of the appropriate lipid lowering medication); 7. Performance Status 0–2 on the WHO scale; 8. adult male or female patients >18 years of age; 9. women of childbearing potential must have had a negative serum or urine pregnancy test 48 hours prior to the administration of the first study treatment; 10. patients were able to give written informed consent, obtained according to local guidelines.

The exclusion criteria included 1. cytotoxic chemotherapy, immunotherapy or radiotherapy within 4 weeks prior to randomization; 2. receipt of treatment with Sandostatin LAR^®^ Depot or any other long-acting somatostatin analog within 2 weeks prior to randomization; 3. hepatic artery embolization within the last 6 months (1 month if there were other sites of measurable disease), or cryoablation of hepatic metastasis within 2 months of randomization; 4. prior therapy with mTOR inhibitors (sirolimus, temsirolimus, or everolimus); 5. known intolerance or hypersensitivity to the drugs used in the trial; 6. uncontrolled diabetes mellitus, as defined by fasting serum glucose >1.5 X ULN; 7. severe and/or uncontrolled medical conditions such as unstable angina pectoris, symptomatic congestive heart failure, myocardial infarction ≤ 6 months prior to randomization, serious uncontrolled cardiac arrhythmia, active or uncontrolled severe infection, cirrhosis, chronic active hepatitis or chronic persistent hepatitis, severely impaired lung function, and chronic treatment with corticosteroids or another immunosuppressive agent; 8. known history of HIV seropositivity; 9. active, bleeding diathesis or being on oral anti-vitamin K medication (except low dose coumadin); 10. history of another primary malignancy ≤ 3 years, with the exception of non-melanoma skin cancer or carcinoma in situ of uterine cervix; and 11. female patients who were pregnant or nursing (lactating), or adults of reproductive potential who were not using effective birth control methods. If barrier contraceptives were being used, these had to be continued throughout the trial by both genders.

### 2.2. Laboratory and Imaging Studies

Full blood counts, biochemical profiling, Chromogranin A measurement (CgA; normal value < 4 nmol/l), and computerized tomography were carried out as standard clinical practice, commonly every 3 months of treatment. Evaluation of the response was carried out locally and centrally at the end of the study. The RECIST criteria were applied to evaluate the responses to treatment, as previously described [16].

### 2.3. Pathology

Paraffin-embedded tissues were centrally revised for the confirmation of the diagnosis and positivity in immunohistochemistry (synaptophysin, CgA, and neuron specific enolase). The Ki67 labeling index was calculated centrally, as previously described [17].

### 2.4. Statistical Analysis

The primary endpoint of the study was to estimate the 15-month PFS rate. According to the Flemings’s single-stage design [18], assuming that the expected 15-month PFS rate will be at least 40% and the minimum acceptable rate 20%, a total of 29 patients provided 80% power to test this hypothesis, with a two-sided alpha of 5%. However, due to the low accrual rate, the study was prematurely terminated, leading to a total of 25 enrolled patients.

The PFS was calculated as the time (in months) from study entry to the date of the first documented disease progression, death from any cause, or last contact (whichever occurred first). OS was measured from the date of study entry to the date of the patient’s death from any cause, with alive patients being censored at the date of last follow-up. TTR was estimated from the date of study entry until the date of best response throughout the study. Time-to-event distributions with corresponding 95% confidence intervals (CI) were estimated with the Kaplan-Meier product limit method. The two-sided log-rank test was used to estimate survival differences between groups of patients. The effect of CgA status (normal: <4 nmol/l vs. abnormal: ≥4 nmol/l) on progression and mortality was estimated with hazard ratios obtained by univariate Cox regression models.

The McNemar’s test was used to estimate the differences in CgA status (normal: <4 nmol/l vs. abnormal: ≥4 nmol/l) between baseline and the last treatment cycle, while the Wilcoxon signed-rank test was performed to detect differences in CgA levels (treated as a continuous variable) between the two time points.

The study was conducted on an intent-to-treat (ITT) basis and therefore all enrolled patients were included in the analysis (N = 25). In addition, the primary endpoint was planned to be estimated in the Per Protocol (PP) population, consisting of all patients that received at least one cycle of treatment with everolimus and did not have major protocol violations. Since all patients received at least one cycle of treatment, and no major protocol violations were reported throughout the study, the primary endpoint was analyzed in the ITT population only. The safety profile of everolimus was assessed in the safety population, consisting of all patients that received at least one dose of the study drug (N = 25). The primary and secondary endpoints of the trial were assessed in the entire study population, including both patients receiving everolimus alone and patients receiving everolimus in combination with octreotide LAR.

All of the tests were two-sided at the 5% level of significance. The data cut-off date for this analysis was 1 March 2019. The SAS version 9.3 (SAS Institute Inc., Cary, NC, USA) and the R studio version 3.5.0 were used for statistical analysis and the generation of plots.

## 3. Results

### 3.1. Patient Characteristics

**Figure 1:**
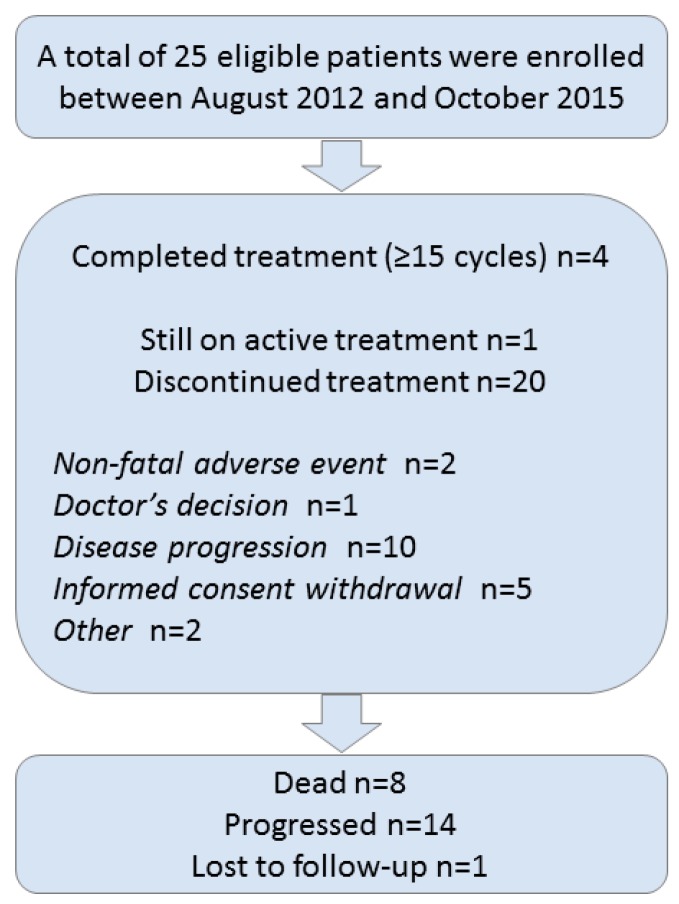
A Remark diagram.

A total of 25 patients (15 women and 10 men) were enrolled in the study between August 2012 and October 2015 (Figure 1). All patients were eligible and no major protocol violations were observed throughout the study. The median age at study entry was 57 years, and most patients had zero performance status (76%) and tumors of intermediate grade (56%), located in the pancreas (40%). Thirteen patients (52%) had undergone surgery. The basic patient and tumor characteristics are summarized in Table 1. Four patients (16%) had locally advanced disease, while 21 patients (84%) presented with metastatic disease at study entry. None of the patients had lung or bone metastases, whereas four patients had metastases in two organs. Three of them had metastases in the liver and lymph nodes (LNS) and one patient had metastases in the liver and the left ovary. A central assessment of Ki67 (by C.P) was available for 17 patients (68%) and confirmed that patients had Ki67 levels lower than 20%, as specified in the inclusion criteria of the protocol.

### 3.2. Chromogranin A Data

**Figure 2:**
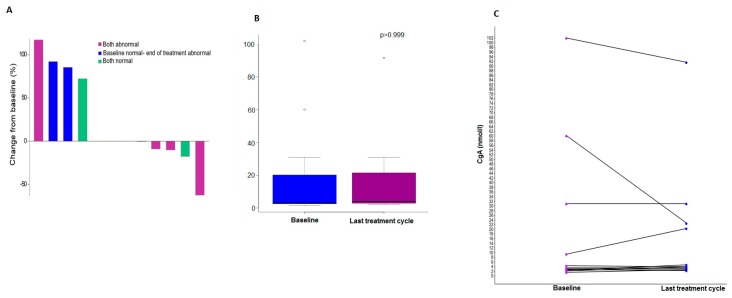
A waterfall plot showing the change from baseline CgA levels at the last cycle of treatment, with (**A**) showing comparative boxplots for CgA levels at baseline and at the last treatment cycle, (**B**) showing the time course of CgA levels at baseline, and (**C**) showing that at the last cycle of treatment per patient.

Of the 25 enrolled patients, 24 had available data regarding the levels of CgA at baseline. The median CgA at baseline was 3.5 nmol/l, while the median CgA value at the last cycle of treatment for the 12 patients with available information was 4 nmol/l (Wilcoxon Signed Rank *p* > 0.999) (Table 2). Levels lower than 4 nmol/l were considered normal, and more than half of the patients (54.2%) had normal levels of CgA at baseline. Of the 12 patients with available CgA levels at baseline and at the last cycle of treatment, five patients (41.7%) had abnormal CgA levels both at the beginning and at the end of treatment, five patients had normal CgA levels at both time points, and only two patients with normal CgA at baseline had abnormal CgA levels at the end of treatment (McNemar’s *p* = 0.16). In three of the 12 patients with CgA information at baseline and at the last cycle, there was no change detected between the two time points, but four patients experienced an increase in the CgA levels, and in five patients, the levels of CgA at the last cycle of treatment were decreased compared to the levels observed at baseline (Figure 2).

### 3.3. Treatment Cycles

All enrolled patients received at least one cycle of everolimus. In total, 324 cycles of treatment (median: 5; range: 1–82) had been administered until the time of the analysis. Four patients completed at least 15 cycles of treatment with everolimus (median: 27; range: 15–59), while 20 patients (80%) discontinued treatment, and one patient was still on active treatment at the cut-off date for this analysis (March 2019). The reasons for treatment discontinuation were non-fatal toxicity (two patients; 10%), the doctor’s decision (one patient; 5%), progression (10 patients; 50%), informed consent withdrawal (five patients; 25%), and other reasons in two patients (6 weeks without therapy due to issues with health insurance, and a second malignancy of urothelial carcinoma of the bladder). Two patients received only one cycle of everolimus and withdrew their informed consent. It is of note that 18 patients (72%) concomitantly received LAR.

### 3.4. Response Assessment

**Figure 3:**
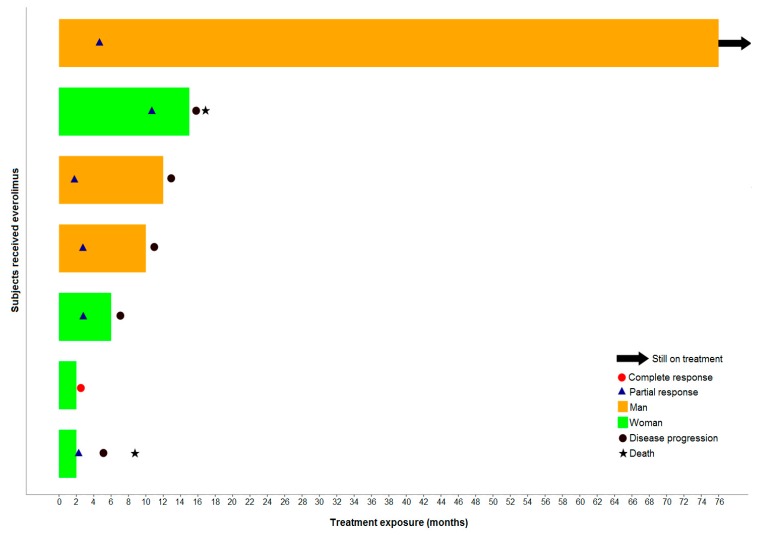
A swimmer plot indicating the time to response for the seven patients with objective responses, according to local tumor assessment. Each bar represents a different patient.

Among the 25 patients enrolled in the study, seven patients (28%) achieved an objective tumor response (one patient with a complete response and six had partial responses), according to the investigators’ assessment in the local hospitals/institutions, in a median of 2.6 months (95% CI 1.8–4.7). Twelve patients had stable disease and four had disease progression. The disease control rate (i.e., the percentage of patients with a complete/partial response, or stable disease as the best response) was 76% according to the local assessment, and was achieved in a median of 2.3 months (95% CI 1.8–2.6). The tumor responses were not assessed for two patients due to treatment discontinuation prior to evaluation. Among the response-evaluable patients (N = 23), the objective response rate was 30.4% and the disease control rate 82.6%, according to the local assessment. Figure 3 presents a time-to-event swimmer plot for the seven patients with an objective response based on the local assessment. The median duration of response for the seven patients who achieved objective responses was 8.5 months (95% CI 3.1-NR), and for the disease control rate was 13.9 months (95% CI 3.9-NR). It is of note that the one patient who achieved a complete response based on the local assessment had normal CgA levels at baseline. Similarly, four of the six patients who achieved partial responses had normal CgA, while only one patient with normal CgA at baseline had progressive disease as the best response, based on the investigators’ assessment. The central radiological assessment according to the RECIST 1.1 criteria was available for 23 patients (92%). Among them, objective responses (partial responses) were observed in 10 patients (43.5%), 11 patients (47.8%) had stable disease, and two (8.7%) had progressive disease. The disease control rate was 91.3%, based on the central assessment. Out of the four patients with locally advanced disease, three achieved objective responses and one patient had stable disease according to the central assessment. Of the 18 patients who received both octreotide LAR and everolimus, nine patients had PR and nine SD at the central radiological assessment.

### 3.5. Survival Analysis

**Figure 4:**
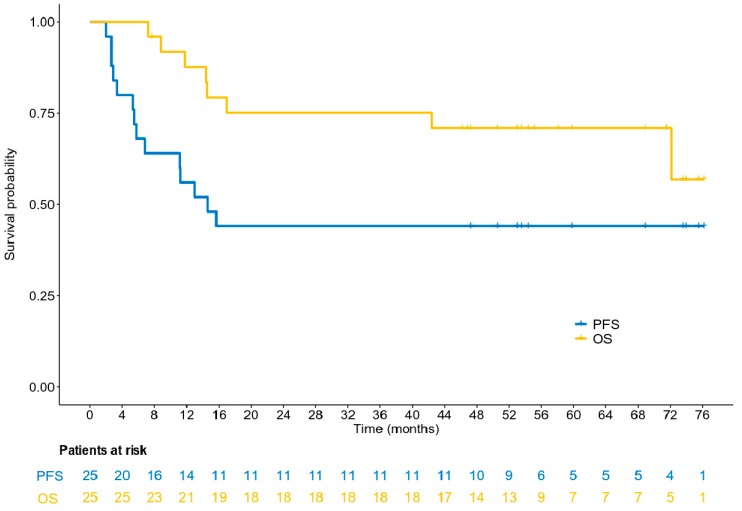
A Kaplan-Meier plot with respect to progression-free survival (PFS) and overall survival (OS).

**Figure 5:**
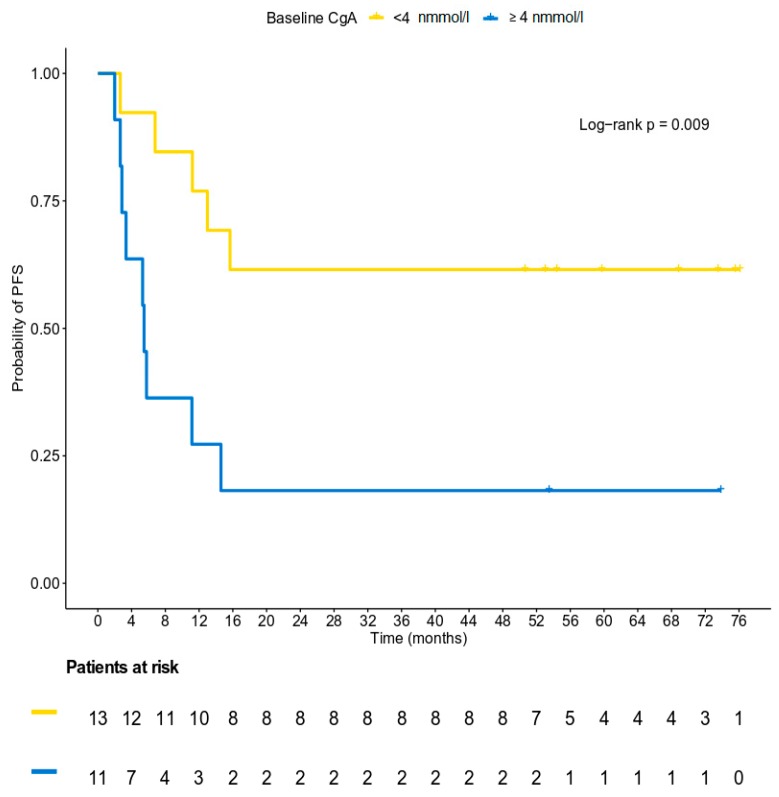
A Kaplan-Meier plot with respect to PFS based on the status of chromogranin levels at baseline.

**Figure 6:**
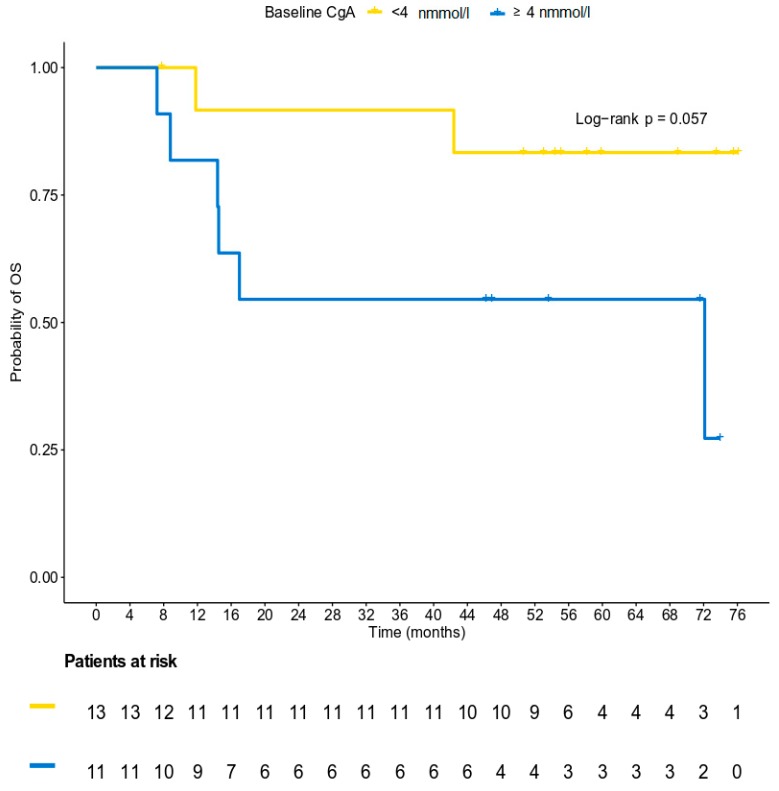
A Kaplan-Meier plot with respect to OS based on the status of chromogranin levels at baseline.

At a median follow-up of 58.1 months (95% CI 50.6–71.6), 14 patients (56%) had experienced disease progression and eight patients (32%) had died. Seven of them died as a result of their tumor, while for one patient, the cause of death was unknown. The median PFS was 14.6 months (95% CI 5.8–15.7), whereas the median OS had not yet been reached at the time of the analysis. The 15-month PFS rate was 48% (95% CI 0.28–0.69) (Figure 4). The median PFS and OS for patients with normal CgA values at baseline had not yet been reached at the time of the analysis, while the median PFS for patients with baseline CgA ≥ 4 nmol/l was 5.5 months (log-rank *p* = 0.009). The 15-month PFS rate for patients with normal and abnormal CgA levels at baseline was 69.2% and 18.2%, respectively (Figure 5). The risk of progression was significantly lower for patients with normal CgA values at baseline (HR = 0.25, 95% CI 0.08–0.77, Wald’s *p* = 0.016), whereas a trend towards a lower risk of death was detected for patients with lower (normal) values of CgA at baseline (HR = 0.24, 95% CI 0.05–1.19, *p* = 0.080, Figure 6). It should be mentioned, however, that the power of these results is limited due to the small number of patients who experienced the events of interest in each category, and the results should be interpreted with caution until confirmed in larger cohorts.

### 3.6. Safety Profile

All 25 patients received at least one dose of everolimus and were, therefore, assessed for safety. A total of 204 adverse events were reported in 24 patients (96%), and most of them were of grade 1–2 (181 events; 88.7%) (Table S1). Twenty-three grade 3–4 events were reported in 14 patients (56%) (20 grade 3 events in 13 patients, 52%; and three grade 4 events in 3 patients, 12%). No toxic deaths were reported. The most commonly reported adverse events belonged to investigations (69 events; 23 patients), followed by metabolism and nutrition disorders (47 events; 18 patients). Nine events of blood and lymphatic disorders were observed in nine patients and were all of grade 1–3. In total, 114 events reported in 22 patients were related to everolimus. Most of them were of grade 1–2 (96 events in 20 patients), while the remaining 18 were events of grade 3–4. Table 3 presents the grade 1 and 2 adverse events in more than 10% of the patients, while Table 4 presents the incidence of treatment related to grade 3–4 events. Among them, two grade 3 events of diarrhea were recorded (in two patients) and three grade 3 events of oral stomatitis (in three patients). In addition, two events of Gamma-Glutamyl Transferase (GGT) increase (one of grade 3 and one of grade 4), one grade 3 event of neutropenia, and one (grade 3) event of anemia were observed. A total of 13 serious adverse events were reported in nine patients (36%). Eight of them were related to treatment with everolimus and included two events of diarrhea (one of grade 2 and one of grade 3), one event (grade 3) of oral stomatitis, one grade 3 infection (bacteremia), one (grade 1) H1N1 pneumonia, one grade 1 fever, and two events of creatinine phosphokinase increase (one event of grade 3 and one of grade 4). It should be mentioned that the grade 3 events of bacteremia and H1N1 pneumonia were detected in the same patient. Similarly, grade 3 diarrhea and the grade 1 fever were observed in one patient.

## 4. Discussion

Herein, we report the results of a phase II study evaluating the efficacy of everolimus as a first-line therapy in treatment-naïve patients with advanced GEP NETs. Our study assessed the radiographic responses in 23 evaluable patients, centrally, and found 43% PR, 48% SD, and 9% PD. The median PFS of all patients was 14.6 months, while the 15-month PFS rate was 48%.

Although not directly comparable, these responses and PFS data are better compared to the previously reported data on single-agent therapy with everolimus, and in the context of more advanced treatment lines. In particular, RADIANT-3 investigated the use of everolimus (10 mg daily) versus placebo in previously pretreated, mostly low-grade, advanced, non-functional pancreatic NETs (pNETs) [7]. The progression free survival (PFS) was 11 months in the everolimus arm, and the objective response rate (ORR) in that study was only 5% in the everolimus arm, underscoring the main benefit of single-agent everolimus in sustaining stable disease, rather than in the radiological response according to RECIST criteria. The RADIANT-4 trial tested the effect of everolimus compared to placebo in pretreated, nonfunctional, non-pancreatic gastrointestinal or lung NETs [6]. Similarly to in RADIANT-2, the PFS was 11 months in the everolimus arm, and the ORR by central radiological assessment was only 2%.

Although it was not an endpoint of our study, an important observation was that of those 18 patients who received both octreotide LAR and everolimus, nine patients had PR and nine had SD at the central radiological assessment. Previous studies have reported the results of combination therapy, including octreotide LAR and everolimus. In particular, RADIANT-2 investigated the use of everolimus (10 mg daily) and the somatostatin analogue (SA) octreotide LAR (30 mg every 28 days) compared to octreotide LAR in advanced low- and intermediate-grade functional GEP NETs and lung NETs, after progression in previous treatments [12]. The final analysis indicated no difference in OS between the two treatment arms, but the heterogeneous study population, unbalanced patient characteristics, uneven SA exposure, and crossover study design have most likely influenced the results [19]. The median PFS in the combination arm was high at 16.4 months, while in the octreotide LAR arm it was 11.3 months. A secondary analysis, based on the previous SA exposure status of patients enrolled in RADIANT-2, indicated that patients in the combination arm without previous SA exposure had a longer median PFS (25.2 months, 95% CI, 12.0–not reached) compared to patients with previous SA exposure (PFS: 14.3 months, 95% confidence interval, CI, 12.0–20.1) [20].

A third phase II study evaluated everolimus in advanced, chemotherapy-pretreated, pancreatic NETs [15]. The study had two strata, based on the co-administration of octreotide LAR. The stratum with combination therapy also reported high responses, with a PR of 4.4% and SD of 80%, indicating a high combinatorial effect of everolimus and octreotide LAR.

A fourth retrospective study of 57 pretreated patients, who had received everolimus with the SA lanreotide, was associated with a PR rate of 15.1%, an SD of 67%, and a median time-to-progression of 25.8 months (95% CI, 11.3, 40.3), adding to the evidence on the efficacy of combination therapy [21].

Most importantly, our study indicated a DCR of 91%. This result can be attributed to the combinatorial effect of targeted therapies (everolimus and SA). Similarly to our study, a previous phase II ITMO study testing everolimus with SA indicated a 92% DCR [22]. Our study is in line with this study on the efficacy of everolimus in NETs, and demonstrates the potential activity of the combination to achieve tumor shrinkage in treatment-naïve NETs, independently from the presence or absence of functioning syndromes. According to the updates on clinical outcomes at 5-year follow-up in the ITMO study, everolimus plus octreotide was shown to be active, with a median OS of 61.0 months (95% CI 49.8–not reached), without major side effects in the long term [23].

All of these data, together with our study, indicate the need for further studies on the use of combination drugs, including everolimus and SA, in the first-line treatment of advanced GEP NETs. The combination of everolimus and octreotide LAR has a high response rate and increases PFS.

The adverse event profile was similar to that of previously reported studies. Among those assessed as related to treatment included stomatitis (all grades: 24%, G3–4: 12%) diarrhea (all grades: 40%, G3–4: 8%) and pneumonitis (all grades: 8%, G3–4: 0%). All of the side effects were reversible, and no deaths were recorded.

In RADIANT-2 and -3, the toxicity of everolimus was low, with grade 3–4 toxicities in 6% of patients, and the most common toxicities being stomatitis, rash, diarrhea, fatigue, and pneumonitis [7,12]. The lower adverse event profile observed in our study could be due to a bias related to the small number of cases, but it could be also related to the experience acquired over the years with this drug and the proactive approach of the physicians in relation to the most common and fairly serious side effects, such as stomatitis, diarrhea, and pneumonitis.

An important adverse event in this trial and the previous phase II and III clinical trials with everolimus is hyperglycemia. This is a multidimensional side effect caused by several factors such as the anatomical integrity of the endocrine pancreas, as in the case of pancreatectomy, and the functional suppression of insulin by SA or everolimus. In our study, the all-grade rate of hyperglycemia was 68% (16 patients), while the G3–4 rate was 4% (one patient). In contrast, this side effect was not recorded for a similar study conducted in Italy [22]. Hyperglycemia, in 10–13% of patients (all grades), was recorded in all Radiant studies and a phase II study, as a side effect of everolimus monotherapy or the administration of everolimus with SA [6,7,12,15]. In none of these studies was the rate of prior surgery and concomitant medications reported, and thus the effect of hyperglycemia was mostly attributed to the employed drugs.

In the COOPERATE phase II trial in advanced pNETs, comparing everolimus with pasireotide versus everolimus, hyperglycemia was 75% (all grades) and 37% (G3–4), versus 27% (all grades) and 11% (G3–4) [24]. This increased glycemic effect could be due to the multiligand SA pasireotide that, compared with octreotide, has a greater binding affinity for SSTR 1,3,5 and exhibits increased IGF1 inhibition [24].

The results of this phase II study are to be interpreted with caution as it had several limitations, two of the most important being the small number of patients included in the study and the fact that this study was not designed to evaluate whether combination therapy is superior to monotherapy.

## 5. Conclusions

The combination of octreotide LAR and everolimus offers a promising first-line treatment option for advanced, well-and moderately-differentiated GEP NETs, with similar toxicity to the single agent everolimus. The results of our study, including 25 patients, warrant further confirmation in a larger study and support the upfront use of combination therapy in these tumors.

## Figures and Tables

**Table 1:** Selected patient and tumor characteristics.

Parameter	Total
	(N = 25)
	Median (min,max)
**Age (in years)** ^	56.9 (37.6,79.9)
**Tumor size (in mm)** *	35.0 (15.0,100.0)
**Ki67 (% of positive nuclei per 2000 cells, central assessment)** *	0.0 (0.0–20.0)
	**N (%)**
**Sex**	
Man	10 (40.0)
Woman	15 (60.0)
**PS**	
0	19 (76.0)
1	6 (24.0)
**Sporadic**	
No	4 (16.0)
Yes	21 (84.0)
**MEN testing**	
No	24 (96.0)
Yes	1 (4.0)
**Initial surgery**	
No	12 (48.0)
Yes	13 (52.0)
**Type of surgery**	
Laparotomy	1 (7.7)
Open surgery	12 (92.3)
**Type of open surgery**	
Total resection	4 (33.3)
Subtotal resection	5 (41.7)
Other **	3 (25.0)
**Tumor localization**	
Jejunum	2 (8.0)
Ileus	4 (16.0)
Colon	2 (8.0)
Pancreas	10 (40.0)
Unknown primary	2 (8.0)
Other ***	5 (20.0)
**Tumor grade**	
G1 [Low grade]	11 (44.0)
G2 [Intermediate grade]	14 (56.0)
**Ki67 (local assessment)**	
<3%	11(44.0)
3–20%	14 (56.0)
**Ki67 (central assessment)** *	
<3%	10 (58.8)
3–20%	7 (41.2)
**Locally advanced** ^	
No	21 (84.0)
Yes	4 (16.0)
**Number of metastatic sites** ^	
0	4 (16.0)
1	17 (68.0)
2	4 (16.0)
**Site of metastases** ^	
Lung	0 (0.0)
Liver	19 (76.0)
Bones	0 (0.0)
LNS	5 (20.0)
Other ****	1 (4.0)

LNS: lymph nodes; * Data not available for all subjects. Missing values: Tumor size = 12, Ki67 (central assessment) = 8; ** One patient underwent subtotal pancreatectomy, splenectomy and liver metastasectomy, one had colectomy and one had biopsy and open-close surgery; *** Three patients had tumors in the small bowel, one in the messenterium and one in the small intestine-terminal ileum; **** Metastasis in the left ovary. ^ at study entry.

**Table 2:** The levels of CgA at baseline and at the last treatment cycle.

CgA	Time point		
	Baseline	Last Cycle	*p*-Value
N	24	12	>0.999 ^
mean (std)	21.22 (30.36)	16.05 (25.73)	
median	3.50	4	
min-max	1.57–102.0	2.30–91.60	
p25–p75	2.55–27.60	2.90-21.50	
Normal (N,%)	13 (54.2)	5 (41.7)	0.16 ^^
Abnormal (N,%)	11 (45.8)	7 (58.3)	

^ Wilcoxon signed-rank test; ^^ McNemar’s test.

**Table 3:** Grade 1 and 2 adverse events occurring in more than 10% of the safety population.

Adverse Event	Grade 1			Grade 2		
System Organ Class	N of evts	N of pts	% pts	N of evts	N of pts	% pts
Preferred Term						
Anemia	5	5	20.00	3	3	12.00
Diarrhea	2	2	8.00	6	6	24.00
Edema limbs	4	4	16.00	1	1	4.00
Fatigue	4	4	16.00	2	2	8.00
Fever	4	4	16.00	1	1	4.00
Alanine aminotransferase increased	6	6	24.00	4	4	16.00
Alkaline phosphatase increased	7	7	28.00	0	0	0.00
Aspartate aminotransferase increased	6	6	24.00	4	4	16.00
Cholesterol high	6	6	24.00	2	2	8.00
Creatinine increased	3	3	12.00	1	1	4.00
GGT increased	2	2	8.00	5	5	20.00
White blood cell decreased	4	4	16.00	1	1	4.00
Hyperglycemia	8	8	32.00	8	8	32.00
Hypertriglyceridemia	5	5	20.00	1	1	4.00
Hypocalcemia	4	4	16.00	1	1	4.00
Hypokalemia	3	3	12.00	1	1	4.00
Hypophosphatemia	2	2	8.00	3	3	12.00
Rash maculo-papular	4	4	16.00	2	2	8.00

N: number; evts: events; pts: patients.

**Table 4:** The incidence of grade 3 and grade 4 adverse events related to treatment with everolimus.

Adverse Event	Grade 3			Grade 4		
System Organ Class	N of evts	N of pts	% pts	N of evts	N of pts	% pts
Preferred Term						
**Total**	16	11	44.00	2	2	8.00
**Blood and lymphatic system disorders**	1	1	4.00	0	0	0.00
Anemia	1	1	4.00	0	0	0.00
**Gastrointestinal disorders**	5	5	20.00	0	0	0.00
Diarrhea	2	2	8.00	0	0	0.00
Mucositis oral	3	3	12.00	0	0	0.00
**Infections and infestations**	2	2	8.00	0	0	0.00
Infections and infestations - Other, specify *	2	2	8.00	0	0	0.00
**Investigations**	4	4	16.00	2	2	8.00
Alanine aminotransferase increased	1	1	4.00	0	0	0.00
CPK increased	1	1	4.00	1	1	4.00
GGT increased	1	1	4.00	1	1	4.00
Neutrophil count decreased	1	1	4.00	0	0	0.00
**Metabolism and nutrition disorders**	3	3	12.00	0	0	0.00
Anorexia	1	1	4.00	0	0	0.00
Hyperglycemia	1	1	4.00	0	0	0.00
Hypokalemia	1	1	4.00	0	0	0.00
**Vascular disorders**	1	1	4.00	0	0	0.00
Hypertension	1	1	4.00	0	0	0.00

N: number; evts: events, pts: patients; * One grade 3 event of bacteremia and one grade 3 event of H1N1 pneumonia.

## Supplementary Materials

The following are available online at https://www.mdpi.com/2079-7737/9/3/51/s1, Table S1: Incidence of grade 1–4 adverse events by grade.

## References

[1] Modlin I.M., Oberg K., Chung D.C., Jensen R.T., de Herder W.W., Thakker R.V., Caplin M., Delle Fave G., Kaltsas G.A., Krenning E.P. (2008). Gastroenteropancreatic neuroendocrine tumours. Lancet Oncol..

[2] Chauhan A., Yu Q., Ray N., Farooqui Z., Huang B., Durbin E.B., Tucker T., Evers M., Arnold S., Anthony L.B. (2018). Global burden of neuroendocrine tumors and changing incidence in Kentucky. Oncotarget.

[3] Dasari A., Shen C., Halperin D., Zhao B., Zhou S., Xu Y., Shih T., Yao J.C. (2017). Trends in the Incidence, Prevalence, and Survival Outcomes in Patients With Neuroendocrine Tumors in the United States. JAMA Oncol..

[4] Caplin M.E., Pavel M., Cwikla J.B., Phan A.T., Raderer M., Sedlackova E., Cadiot G., Wolin E.M., Capdevila J., Wall L. (2014). Lanreotide in metastatic enteropancreatic neuroendocrine tumors. N. Engl. J. Med..

[5] Raymond E., Dahan L., Raoul J.L., Bang Y.J., Borbath I., Lombard-Bohas C., Valle J., Metrakos P., Smith D., Vinik A. (2011). Sunitinib malate for the treatment of pancreatic neuroendocrine tumors. N. Engl. J. Med..

[6] Yao J.C., Fazio N., Singh S., Buzzoni R., Carnaghi C., Wolin E., Tomasek J., Raderer M., Lahner H., Voi M. (2016). Everolimus for the treatment of advanced, non-functional neuroendocrine tumours of the lung or gastrointestinal tract (RADIANT-4): A randomised, placebo-controlled, phase 3 study. Lancet.

[7] Yao J.C., Shah M.H., Ito T., Bohas C.L., Wolin E.M., Van Cutsem E., Hobday T.J., Okusaka T., Capdevila J., de Vries E.G. (2011). Everolimus for advanced pancreatic neuroendocrine tumors. N. Engl. J. Med..

[8] Pyronnet S., Bousquet C., Najib S., Azar R., Laklai H., Susini C. (2008). Antitumor effects of somatostatin. Mol. Cell Endocrinol..

[9] Rinke A., Muller H.H., Schade-Brittinger C., Klose K.J., Barth P., Wied M., Mayer C., Aminossadati B., Pape U.F., Blaker M. (2009). Placebo-controlled, double-blind, prospective, randomized study on the effect of octreotide LAR in the control of tumor growth in patients with metastatic neuroendocrine midgut tumors: A report from the PROMID Study Group. J. Clin. Oncol..

[10] Merola E., Panzuto F., Delle Fave G. (2017). Antiproliferative effect of somatostatin analogs in advanced gastro-entero-pancreatic neuroendocrine tumors: A systematic review and meta-analysis. Oncotarget.

[11] DeBerardinis R.J., Lum J.J., Hatzivassiliou G., Thompson C.B. (2008). The biology of cancer: Metabolic reprogramming fuels cell growth and proliferation. Cell Metab..

[12] Pavel M.E., Hainsworth J.D., Baudin E., Peeters M., Horsch D., Winkler R.E., Klimovsky J., Lebwohl D., Jehl V., Wolin E.M. (2011). Everolimus plus octreotide long-acting repeatable for the treatment of advanced neuroendocrine tumours associated with carcinoid syndrome (RADIANT-2): A randomised, placebo-controlled, phase 3 study. Lancet.

[13] Bousquet C., Lasfargues C., Chalabi M., Billah S.M., Susini C., Vezzosi D., Caron P., Pyronnet S. (2012). Clinical review: Current scientific rationale for the use of somatostatin analogs and mTOR inhibitors in neuroendocrine tumor therapy. J. Clin. Endocrinol. Metab..

[14] Yao J.C., Phan A.T., Chang D.Z., Wolff R.A., Hess K., Gupta S., Jacobs C., Mares J.E., Landgraf A.N., Rashid A. (2008). Efficacy of RAD001 (everolimus) and octreotide LAR in advanced low- to intermediate-grade neuroendocrine tumors: Results of a phase II study. J. Clin. Oncol..

[15] Yao J.C., Lombard-Bohas C., Baudin E., Kvols L.K., Rougier P., Ruszniewski P., Hoosen S., St Peter J., Haas T., Lebwohl D. (2010). Daily oral everolimus activity in patients with metastatic pancreatic neuroendocrine tumors after failure of cytotoxic chemotherapy: A phase II trial. J. Clin. Oncol..

[16] Schwartz L.H., Litiere S., de Vries E., Ford R., Gwyther S., Mandrekar S., Shankar L., Bogaerts J., Chen A., Dancey J. (2016). RECIST 1.1-Update and clarification: From the RECIST committee. Eur. J. Cancer.

[17] Kloppel G., La Rosa S. (2018). Ki67 labeling index: Assessment and prognostic role in gastroenteropancreatic neuroendocrine neoplasms. Virchows Arch..

[18] Fleming T.R. (1982). One-sample multiple testing procedure for phase II clinical trials. Biometrics.

[19] Pavel M.E., Baudin E., Oberg K.E., Hainsworth J.D., Voi M., Rouyrre N., Peeters M., Gross D.J., Yao J.C. (2017). Efficacy of everolimus plus octreotide LAR in patients with advanced neuroendocrine tumor and carcinoid syndrome: Final overall survival from the randomized, placebo-controlled phase 3 RADIANT-2 study. Ann. Oncol..

[20] Anthony L.B., Pavel M.E., Hainsworth J.D., Kvols L.K., Segal S., Horsch D., Van Cutsem E., Oberg K., Yao J.C. (2015). Impact of Previous Somatostatin Analogue Use on the Activity of Everolimus in Patients with Advanced Neuroendocrine Tumors: Analysis from the Phase III RADIANT-2 Trial. Neuroendocrinology.

[21] Capdevila J., Sevilla I., Alonso V., Anton Aparicio L., Jimenez Fonseca P., Grande E., Reina J.J., Manzano J.L., Alonso Lajara J.D., Barriuso J. (2015). Evaluation of the efficacy and safety of lanreotide in combination with targeted therapies in patients with neuroendocrine tumours in clinical practice: A retrospective cross-sectional analysis. BMC Cancer.

[22] Bajetta E., Catena L., Fazio N., Pusceddu S., Biondani P., Blanco G., Ricci S., Aieta M., Pucci F., Valente M. (2014). Everolimus in combination with octreotide long-acting repeatable in a first-line setting for patients with neuroendocrine tumors: An ITMO group study. Cancer.

[23] Bajetta E., Catena L., Pusceddu S., Spada F., Iannacone C., Sarno I., Di Menna G., Dottorini L., Marte A.M. (2018). Everolimus in Combination with Octreotide Long-Acting Repeatable in a First-Line Setting for Patients with Neuroendocrine Tumors: A 5-Year Update. Neuroendocrinology.

[24] Kulke M.H., Ruszniewski P., Van Cutsem E., Lombard-Bohas C., Valle J.W., De Herder W.W., Pavel M., Degtyarev E., Brase J.C., Bubuteishvili-Pacaud L. (2017). A randomized, open-label, phase 2 study of everolimus in combination with pasireotide LAR or everolimus alone in advanced, well-differentiated, progressive pancreatic neuroendocrine tumors: COOPERATE-2 trial. Ann. Oncol..

