# Supplementary material for: Efficacy and Safety of First-Line Everolimus Therapy Alone or in Combination with Octreotide in Gastroenteropancreatic Neuroendocrine Tumors. A Hellenic Cooperative Oncology Group (HeCOG) Study
Source: Biology (Basel). 2020 Mar 9;9(3):51. doi: 10.3390/biology9030051 (PMC7150771; doi:10.3390/biology9030051)
Supplement: Supplementary file 1 [file biology-09-00051-s001.pdf]

Supplemental Table 1. Incidence of grade 1-4 adverse events by grade.

	Grade 1			Grade 2			Grade 3			Grade 4		
**System Organ Class**<br>**Preferred Term**	**N of evts**	**N of pts**	**% pts**	**N of evts**	**N of pts**	**% pts**	**N of evts**	**N of pts**	**% pts**	**N of evts**	**N of pts**	**% pts**
**Total**	118	24	96.00	63	23	92.00	20	13	52.00	3	3	12.00
**Blood and lymphatic system disorders**	5	5	20.00	3	3	12.00	1	1	4.00	0	0	0.00
Anemia	5	5	20.00	3	3	12.00	1	1	4.00	0	0	0.00
Eye disorders	1	1	4.00	0	0	0.00	0	0	0.00	0	0	0.00
Conjunctivitis	1	1	4.00	0	0	0.00	0	0	0.00	0	0	0.00
**Gastrointestinal disorders**	8	5	20.00	8	8	32.00	5	5	20.00	0	0	0.00
Abdominal pain	1	1	4.00	0	0	0.00	0	0	0.00	0	0	0.00
Colitis	1	1	4.00	0	0	0.00	0	0	0.00	0	0	0.00
Diarrhea	2	2	8.00	6	6	24.00	2	2	8.00	0	0	0.00
Dyspepsia	1	1	4.00	0	0	0.00	0	0	0.00	0	0	0.00
Mucositis oral	2	2	8.00	1	1	4.00	3	3	12.00	0	0	0.00
Nausea	1	1	4.00	0	0	0.00	0	0	0.00	0	0	0.00
Vomiting	0	0	0.00	1	1	4.00	0	0	0.00	0	0	0.00
**General disorders and administration site conditions**	13	12	48.00	4	3	12.00	0	0	0.00	0	0	0.00
Edema limbs	4	4	16.00	1	1	4.00	0	0	0.00	0	0	0.00
Fatigue	4	4	16.00	2	2	8.00	0	0	0.00	0	0	0.00
Fever	4	4	16.00	1	1	4.00	0	0	0.00	0	0	0.00
Flu like symptoms	1	1	4.00	0	0	0.00	0	0	0.00	0	0	0.00
**Immune system disorders**	0	0	0.00	0	0	0.00	1	1	4.00	0	0	0.00
Allergic reaction	0	0	0.00	0	0	0.00	1	1	4.00	0	0	0.00
**Infections and infestations**	3	2	8.00	1	1	4.00	3	3	12.00	0	0	0.00
Biliary tract infection	0	0	0.00	0	0	0.00	1	1	4.00	0	0	0.00
Infections and infestations - Other, specify*	1	1	4.00	0	0	0.00	2	2	8.00	0	0	0.00
Papulopustular rash	1	1	4.00	0	0	0.00	0	0	0.00	0	0	0.00
Upper respiratory infection	1	1	4.00	0	0	0.00	0	0	0.00	0	0	0.00
Urinary tract infection	0	0	0.00	1	1	4.00	0	0	0.00	0	0	0.00
**Injury, poisoning and procedural complications**	0	0	0.00	1	1	4.00	0	0	0.00	0	0	0.00
Injury, poisoning and procedural complications - Other, specify**	0	0	0.00	1	1	4.00	0	0	0.00	0	0	0.00

**Investigations**	41	19	76.00	20	14	56.00	5	5	20.00	3	3	12.00
Alanine aminotransferase increased	6	6	24.00	4	4	16.00	1	1	4.00	0	0	0.00
Alkaline phosphatase increased	7	7	28.00	0	0	0.00	1	1	4.00	0	0	0.00
Aspartate aminotransferase increased	6	6	24.00	4	4	16.00	0	0	0.00	0	0	0.00
CPK increased	0	0	0.00	0	0	0.00	1	1	4.00	1	1	4.00
Cholesterol high	6	6	24.00	2	2	8.00	0	0	0.00	0	0	0.00
Creatinine increased	3	3	12.00	1	1	4.00	0	0	0.00	0	0	0.00
GGT increased	2	2	8.00	5	5	20.00	1	1	4.00	2	2	8.00
Investigations - Other, specify***	2	2	8.00	1	1	4.00	0	0	0.00	0	0	0.00
Neutrophil count decreased	1	1	4.00	1	1	4.00	1	1	4.00	0	0	0.00
Platelet count decreased	2	2	8.00	0	0	0.00	0	0	0.00	0	0	0.00
Weight loss	2	2	8.00	1	1	4.00	0	0	0.00	0	0	0.00
White blood cell decreased	4	4	16.00	1	1	4.00	0	0	0.00	0	0	0.00
**Metabolism and nutrition disorders**	28	15	60.00	15	10	40.00	4	3	12.00	0	0	0.00
Anorexia	0	0	0.00	1	1	4.00	1	1	4.00	0	0	0.00
Hyperglycemia	8	8	32.00	8	8	32.00	1	1	4.00	0	0	0.00
Hyperkalemia	1	1	4.00	0	0	0.00	0	0	0.00	0	0	0.00
Hypertriglyceridemia	5	5	20.00	1	1	4.00	0	0	0.00	0	0	0.00
Hyperuricemia	1	1	4.00	0	0	0.00	0	0	0.00	0	0	0.00
Hypoalbuminemia	2	2	8.00	0	0	0.00	0	0	0.00	0	0	0.00
Hypocalcemia	4	4	16.00	1	1	4.00	0	0	0.00	0	0	0.00
Hypoglycemia	1	1	4.00	0	0	0.00	0	0	0.00	0	0	0.00
Hypokalemia	3	3	12.00	1	1	4.00	1	1	4.00	0	0	0.00
Hypomagnesemia	1	1	4.00	0	0	0.00	1	1	4.00	0	0	0.00
Hypophosphatemia	2	2	8.00	3	3	12.00	0	0	0.00	0	0	0.00
**Nervous system disorders**	1	1	4.00	2	2	8.00	0	0	0.00	0	0	0.00
Dysgeusia	0	0	0.00	1	1	4.00	0	0	0.00	0	0	0.00
Headache	0	0	0.00	1	1	4.00	0	0	0.00	0	0	0.00
Peripheral sensory neuropathy	1	1	4.00	0	0	0.00	0	0	0.00	0	0	0.00
**Renal and urinary disorders**	2	2	8.00	1	1	4.00	0	0	0.00	0	0	0.00
Proteinuria	2	2	8.00	1	1	4.00	0	0	0.00	0	0	0.00
**Respiratory, thoracic and mediastinal disorders**	5	4	16.00	2	2	8.00	0	0	0.00	0	0	0.00
Cough	2	2	8.00	1	1	4.00	0	0	0.00	0	0	0.00
Epistaxis	1	1	4.00	0	0	0.00	0	0	0.00	0	0	0.00

Pleural effusion	1	1	4.00	0	0	0.00	0	0	0.00	0	0	0.00
Pneumonitis	1	1	4.00	1	1	4.00	0	0	0.00	0	0	0.00
**Skin and subcutaneous tissue disorders**	10	8	32.00	4	4	16.00	0	0	0.00	0	0	0.00
Dry skin	1	1	4.00	0	0	0.00	0	0	0.00	0	0	0.00
Nail loss	0	0	0.00	1	1	4.00	0	0	0.00	0	0	0.00
Palmar-plantar erythrodysesthesia syndrome	0	0	0.00	1	1	4.00	0	0	0.00	0	0	0.00
Pruritus	1	1	4.00	0	0	0.00	0	0	0.00	0	0	0.00
Rash acneiform	1	1	4.00	0	0	0.00	0	0	0.00	0	0	0.00
Rash maculo-papular	4	4	16.00	2	2	8.00	0	0	0.00	0	0	0.00
Skin and subcutaneous tissue disorders - Other, specify****	2	2	8.00	0	0	0.00	0	0	0.00	0	0	0.00
Skin hypopigmentation	1	1	4.00	0	0	0.00	0	0	0.00	0	0	0.00
**Vascular disorders**	1	1	4.00	2	2	8.00	1	1	4.00	0	0	0.00
Hypertension	1	1	4.00	1	1	4.00	1	1	4.00	0	0	0.00
Thromboembolic event	0	0	0.00	1	1	4.00	0	0	0.00	0	0	0.00

N: number; evts: events; pts: patients

*One grade 1 event of viral infection (upper respiratory), one grade 3 event of bacteremia and one grade 3 event of H1N1 pneumonia.

**One grade 2 event of wound abscess.

*** Two grade 1 and one grade 2 events of LDH.

**** One event of nail dystrophia (grade 1) and one of rash hair head (grade 1).
